# The Diagnosis and Treatment of Movable Kidney

**Published:** 1894-01

**Authors:** J. B. S. Holmes

**Affiliations:** Atlanta, Ga.


ATLANTA
MEDICAL AND SURGICAL JOURNAL.

VOL. X. JANUARY, 1894. No. 11.

EDITED BY
LUTHER B. GRANDY, M. D., AND MILLER B. HUTCHINS, M. D.,
WITH THE CO-OPERATION OF
H. V. M. MILLER, M. D., LL. D., VIRGIL O. HARDON, M. D.,
FLOYD W. McRAE, M. D., AND ARTHUR G. HOBBS, M. D.

### *ORIGINAL COMMUNICATIONS.*

### THE DIAGNOSIS AND TREATMENT OF MOVABLE KIDNEY.*

BY J. B. S. HOLMES, M. D.,
ATLANTA, GA.

At the request of our esteemed President I submit this paper, he having suggested the subject. I will not enter into the anatomy of the kidneys, but briefly state their normal position, which is about two inches above the iliac crest for the left and not quite so high for the right. Their upper extremity is about opposite the upper border of the twelfth dorsal vertebra and the lower on a level with the third lumbar; the right, as above mentioned, being normally a little lower than the left. They are, in their normal condition, retro-peritoneal, though in some cases surrounded by the peritoneum. The movable kidney, strictly speaking, being always retro-peritoneal, the floating kidney entirely invested by the peri-

*Read before the Tri-State Medical Association at Chattanooga, Tennessee, October, 1893.

toneum, this being the distinction in the two terms. They are held in their normal position by their fatty capsules, the peritoneum and blood vessels. Movable kidneys exist much oftener than is usually supposed. Statistics show one to every two hundred and fifty subjects. It is found in women more frequently than men, in proportion of seven to one. It also occurs seven times with the right kidney to one with the left. In many undoubted cases of movable kidney no inconvenience whatever is felt and the condition is frequently accidentally discovered by the subject, often producing much alarm and anxiety, fearing a tumor of some serious condition exists. This is especially the case with patients that have thin abdominal walls, where the supposed tumor can be unmistakably felt. Under these circumstances the mental effect is sometimes much greater than the physical disturbance. Here a careful diagnosis must be made. The physician cannot afford to make a mistake between a condition of things that is sometimes attended with little or no inconvenience, and a condition that may be very serious. If he can assure his patient that the lump or supposed tumor is only a movable kidney all fear is dispelled. In many cases, however, great discomfort, inconvenience and broken health is attributed directly to movable kidneys. Many cases of abdominal discomfort, increased by every movement of the patient, where we are frequently at a loss to account for the trouble, can be easily traced to these sources. When turning in bed something is often felt to move in the abdomen, producing a dragging sensation, sometimes giving rise to acute pain. The pain is frequently accompanied by soreness and tenderness over the entire abdominal region; it is occasionally localized in the lumbar region, again extending down the thighs and legs; in some cases producing severe gastralgia and in others intercostal neuralgia. The digestive process is almost always interfered with, in some instances producing nervous dyspepsia with great emaciation. Dilatation of the stomach has been attributed to the pressure of the kidney on the duodenum. Swelling of the lower extremities can sometimes be traced also to the pressure of the misplaced kidney on the ascending vena-cava; and pressure of the kidney on the descending colon sometimes produces severe constipation. After a sudden movement the patient may be attacked with severe abdominal pain, with great tenderness in the region of the kidney, accompanied by rigors, nausea and vomiting. The urine is usually reduced in quantity and dark in color. After a few days there may be a free flow of urine containing pus. The cause of this is probably due to torsion of the ureter and compression, causing hydro-nephrosis or even pyo-nephrosis.

The importance of movable kidney as a factor in gynecological symptoms is often overlooked and not properly appreciated, and it is these that I wish especially to emphasize. The specialist is too prone to attribute every reflex phenomena to disease of the particular organs that he treats. The gynecologist is no exception to the rule. We are too much disposed to attribute every symptom to disease of the ovary, tube or uterus. We get in the habit of putting as much on these organs, especially the ovaries, as our brother, the general practitioner, does on the liver. As a gynecologist, I do insist upon a broader scope of examination. We must not only make a thorough examination of the pelvic organs, but look further. In every case the gynecologist should make a thorough bimanual examination of the abdomen before a diagnosis of the pelvic disease is made. I have under observation at this time a lady from whom I removed, in June last, both ovaries. They were cystic, one as large as an orange. A careful examination before the operation, revealed a movable kidney. I was in position to tell her that the removal of the cystic ovaries would give her great relief but not cure her, that the kidney would have to be anchored, which I will do at an early date, before I could promise her complete relief. She is much benefited, but not entirely cured. Women with misplaced kidneys often suffer with dysmenorrhea and increased nervous disturbance and pain during the menstrual period; hysterical manifestations and often hystero-epilepsy, headache, constipation and gastric disturbances; irritability of the bladder, with painful micturition, is not uncommon. Sometimes there are interruptions to the flow of the urine and occasionally complete suppression, lasting from twelve to twenty-four hours, or longer and due, no doubt, to twisting of the ureters, for both kidneys are sometimes misplaced.

Pain in the ovaries, bearing down sensations with general pelvic pain and discomfort are among the reflex symptoms and might be regarded, without a searching examination, as due entirely to disease in the pelvic organs. For positive diagnosis of misplaced kidneys, we must rely solely upon bimanual palpation.

In patients with thick abdominal walls it is not always an easy matter to detect the kidney, even if it be freely movable. The subjects, however, are usually more or less emaciated, and this aids us greatly in our examination. Where we have reason to suspect a movable kidney, we should always give a free purge, and I prefer one of castor oil and spirits of turpentine. This may be supplemented by a high enema of ox-gall dissolved in hot water. We are then sure to remove all fecal matter and to empty the bowels also of their gaseous contents. With one hand pressed well into the loin, if movable kidney exists, a reniform tumor will often be detected through the abdominal wall with the other. If it be a kidney, it will slip to and fro between the hands.

If you fail to find it, have your patient to assume an erect position, while you sit, in examining for the right kidney, at the right side. Let him incline forward and twist the shoulder from side to side. Press your left hand firmly into the right loin in the normal position of the kidney, at the same time making firm pressure under the free border of the ribs with the finger of the right hand, then bring the fingers of the two hands together. Then have your patient to lie down and elevate his knees, all the while keeping up firm pressure with the thumb of the left hand under the free border of the ribs; then carry your right hand downward over the right side of the abdomen and you will be apt to find the kidney, if it is misplaced. You will detect it as it slips back in position.

Of course in examining for the left kidney the above order should be reversed. Pressure on the kidney will always, without exception, produce nausea and a feeling of faintness. This is an important point in diagnosis. These symptoms, in connection with the shape of the tumor, make the diagnosis almost unmistakable. When the kidney is misplaced an abnormal resonance is always present over its normal location. Fecal masses may be confounded with it, but these can be easily removed and all doubt cleared up by free purgation. Enlargement or tumors of other abdominal organs are apt to present the shape of the misplaced kidney, nor will pressure upon them give rise to the nausea and faintness that always occurs when the kidney is pressed upon. Ovarian and uterine tumors, as a rule, can be easily detected by vaginal and rectal examination. The diagnosis cannot be made between a floating and a movable kidney, though the mobility of the former is greater while the same constitutional symptoms exist in each. For surgical purposes it is, however, desirable to know which we have to contend with. When the diagnosis of movable kidney is clearly made, if no unpleasant symptoms exist, it is well enough to leave your patient alone and advise no treatment of the kidney itself. All violent exercise, such as running, jumping and horseback riding should be interdicted, and if the patient should be a female she should be especially quiet during menstruation, and tight lacing should at all times be avoided. The bowels should be regulated so as to prevent straining at stool. Tonics, judicious exercise in open air, good food and in fact everything tending to improve and build up the system should be advised. Of course should any ill effect be produced upon the general health the kidney should at once be replaced, which can, as a general thing, be done with little or no trouble, and efforts should be made to hold it in position by pads and bandages, which I, however, have little or no faith in. An air pad, held in position by a light flannel binder, I believe would answer a better purpose and produce less discomfort to the patient than almost any other apparatus. An abdominal supporter to hold up the intestines and cause them to support the kidneys would doubtless do good and might be tried. In cases of traumatic displacement we may often effect a cure by replacing the organ, keeping the patient in bed on the back and holding the kidney in place by suitable apparatus. When the symptoms previously described as indicating strangulation occur, the patient should at once be put to bed, replacement attempted and hot baths be given and anodynes used to a limited extent. Should the symptoms continue violent and threatening, I would, if necessary, unhesitatingly resort to surgical procedure. In cases where mechanical devices fail to give relief, if the symptoms are aggravated, the patient's health broken down and he incapacitated for the ordinary duties of life I would unhesitatingly advise surgical procedure. This, it is needless for me to add, should be done under the *strictest* aseptic and antiseptic precautions.

Nephrorrhaphy or suturing the kidney to the structures around it should be done, the patient should be thoroughly purged and the surface rendered aseptic by washing it with green soap, then ether, then a bichloride solution of 1 to 1000. He should be placed with the face downward, inclined to the side and an incision made two and one-half to three inches from the vertebral spines and parallel to them; beginning one-half an inch to one inch below the twelfth rib and extending to near the iliac crest, cut down through the tissues to the capsule of the kidney. Some surgeons advise dividing this and stitching the edges to either side of the incision, while others advise passing the sutures into the kidney substance. This I would not do unless the capsule was too lax and allowed the kidney too much play; then I would not hesitate to pass my suture a slight depth into the kidney itself. It is well to scratch the capsule with your aseptic finger in order to excite plastic exudation. I would recommend for sutures silkworm gut or kangaroo tendon, as the catgut is absorbed too quickly. All bleeding vessels should be closed either by forcible pressure or ligation. Wash the wound thoroughly with hot sterilized water; pass a large size rubber drainage tube to the bottom of the wound. The wound should now be closed with silkworm gut sutures, which I use for all my surgical work. If there should be much oozing, I would place the drainage tube in position, pack the wound with iodoform gauze, place my sutures in position but not tighten them until the oozing had entirely ceased, then the gauze should be removed and stitches tightened. The usual dressing of iodoform gauze should then be applied and the patient kept in bed for three or four weeks, placed upon light diet, the bowels kept well open and the dressings changed when needed to keep them in an aseptic condition. Should strangulation exist, producing hydro- or pyo-nephrosis, the kidney should be opened and drained. Should fixation not be complete and the continued symptoms be grave enough to demand it, I would advise nephrectomy by the lumbar method, as this can be made an extra-peritoneal operation and therefore much safer than by the abdominal method. In case, however, of the floating kidney the abdominal method might be best. In doing the lumbar operation the incision should be made as in the operation for nephrorrhaphy. If the kidney is large a T or V shaped incision may be necessary. Too much traction should not be made upon the kidney, lest when the pedicle is tied it may retract and cause the ligature to slip and perhaps fatal hemorrhage result. The vein and artery should be included in one ligature and the ureter in the other. The blood vessels should not be tied too near to either the aorta or vena-cava, for fear that the thrombus forming in them might slip into the large vessels and produce death. If the peritoneum should be opened it should be closed at once with fine catgut sutures. The wound is now washed and treated as in the method advised in nephrorrhaphy. Nephrectomy should not be performed unless we are satisfied that another kidney exists and is in healthy condition. If an analysis of the urine should show severe disease in the kidney, the movable organ should not be extirpated, unless we have reason to know the other is sound. This can be determined very easily in women, but with greater difficulty in men, by catheterizing the ureters and analyzing the urine of the two separately, and by this means tell which kidney is healthy.

The dressing and after treatment is practically the same after nephrectomy as after nephrorrhaphy.

---

IN order to enable State medical societies to send instructions as to their action upon the matter referred to them by the American Medical Association at its recent meeting at Milwaukee, and for other reasons, the time of meeting of the association at San Francisco has been changed from the first Tuesday in May to the first Tuesday in June, 1894.

